# Final Destination? Pinpointing *Hyella disjuncta* sp. nov. PCC 6712 (Cyanobacteria) Based on Taxonomic Aspects, Multicellularity, Nitrogen Fixation and Biosynthetic Gene Clusters

**DOI:** 10.3390/life11090916

**Published:** 2021-09-03

**Authors:** Patrick Jung, Paul M. D’Agostino, Katharina Brust, Burkhard Büdel, Michael Lakatos

**Affiliations:** 1 Department of Integrative Biotechnology, University of Applied Sciences Kaiserslautern, Carl-Schurz-Str. 10–16, 66953 Pirmasens, Germany; michael.lakatos@hs-kl.de; 2 Department of Technical Biochemistry, Technical University of Dresden, Bergstr. 66, 01069 Dresden, Germany; paul.dagostino@tu-dresden.de; 3 Department of Ecology, University of Kaiserslautern, Erwin Schrödinger Str. 14, 67663 Kaiserslautern, Germany; katharina.brust@web.de; 4 Department of Plant Ecology and Systematics, University of Kaiserslautern, Erwin-Schrödinger Str. 52, 67663 Kaiserslautern, Germany; buedel@bio.uni-kl.de

**Keywords:** Pleurocapsales, genomics, biosynthetic gene cluster, *Chroococcidiopsis*, *Hyella*

## Abstract

Unicellular cyanobacteria inhabit a wide range of ecosytems and can be found throughout the phylum offering space for taxonomic confusion. One example is strain PCC 6712 that was described as *Chlorogloea* sp. (Nostocales) and later assigned to the genus *Chroococcidiopsis* (Chroococcidiopsidales). We now show that this strain belongs to the order Pleurocapsales and term it *Hyella disjuncta* based on morphology, genome analyses and 16S-23S ITS rRNA phylogeny. Genomic analysis indicated that *H. disjuncta* PCC 6712 shared about 44.7% orthologue genes with its closest relative *H. patelloides*. Furthermore, 12 cryptic biosynthetic gene clusters (BGCs) with potential bioactivity, such as a mycosporine-like amino acid BGC, were detected. Interestingly, the full set of nitrogen fixation genes was found in *H. disjuncta* PCC 6712 despite its inability to grow on nitrogen-free medium. A comparison of genes responsible for multicellularity was performed, indicating that most of these genes were present and related to those found in other cyanobacterial orders. This is in contrast to the formation of pseudofilaments—a main feature of the genus *Hyella*—which is weakly expressed in *H. disjuncta* PCC 6712 but prominent in *Hyella patelloides* LEGE 07179. Thus, our study pinpoints crucial but hidden aspects of polyphasic cyanobacterial taxonomy.

## 1. Introduction

Pleurocapsalean cyanobacteria represent a phylogenetically well-framed group (see, e.g., [1,2]) but are still poorly investigated on several levels. Most are unicellular and marked by the formation of baeocytes or pseudofilaments. The problem is that a large number of genera in this order lack sequence data and are very difficult to transfer in pure cultures [1]. Although the order is highly diverse and species rich, only a few genera have been studied to an extent where a highly accurate taxonomic placement based on modern standards has been achieved. This is the case for the genus *Pleurocapsa*, for which in 2019 the neotype and epitype for *P. minor* and *P. fuliginosa* were finally introduced [3]. In order to further strengthen taxonomy within the Pleurocapsales, additional strains need to be revised whose classification were based primarily on morphology, and therefore, they may be incorrect. 

Several names given to species in the order Chroococcidiopsidales such as *Gloeocapsopsis pleurocapsoides* still indicate the difficulties discriminating members of the two orders Chroococcidiopsidales and Pleurocapsales based on morphology, although their 16S rRNA-based phylogeny clearly separates both orders [4]. The most prominent case highlighting this issue is the cyanobacterial strain PCC 6712 that was isolated in 1967 by R. Kunisawa from a freshwater reservoir in Marin County, California and originally reported as *Chlorogloea* sp. (Nostocales) by Kenyon in 1972 [5]. Later, DNA of the strain PCC 6712 was partially sequenced and phylogenetically compared resulting in a reclassification of the strain as *Chroococcidiopsis* sp. (Chroococcidiopsidales) by Waterbury [6]. This decision was also supported by findings of non-motile baeocytes in strain PCC 6712, a clear indicator at that time for this isolate belonged to the order Chroococcidiopsidales because most baeocytous Pleurocapsales have motile baeocytes [7]. However, simultaneously, the authors noticed significant differences between PCC 6712 and other members of the genus *Chroococcidiopsis*. From then on, several studies covering broad scientific fields used this strain, always referring to it as ‘*Chroococcidiopsis* sp.’ PCC 6712, but at the same time, again, reporting striking differences between this strain and other *Chroococcidiopsis* species. This includes early studies on comparative cyanobacterial analyses [8], secondary metabolites [9], fatty acid composition [10], phylogenetic and morphological investigations [4], functional genomics [11], salt tolerance [12] and testing cell counting algorithms [13]. 

The isolate ‘*Chroococcidiopsis* sp.’ PCC 6712 was also a major focus with respect to novel natural products [14] enabled by the release of the full genome by [15]. Brito et al. [14] showed a direct phylogenetic relation between ‘*Chroococcidiopsis* sp.’ PCC 6712 and *Hyella patelloides* LEGE 07179 on a genome level, and a previous study by Brito et al., in 2017 [16], also indicated that the strain is Pleurocapsalean based on 16S phylogeny and might be an additional species of the genus *Hyella*. Interestingly, a discrepancy appeared behind this idea because a main feature of the genus *Hyella* is the prominent formation of pseudofilaments, a characteristic that is well present in *H. patelloides* LEGE 07179 but was not described previously for strain PCC 6712 [6,7,17,18,19].

In the present article, we resolve this situation and apply a full taxonomic investigation including the polyphasic approach as the standard in cyanobacterial taxonomy. This is based on morphological investigations of the strain *Chroococcidiopsis* sp. PCC 6712, including various media and substrates as well as sequencing and phylogenetic analysis of the 16S-23S rRNA. Taxonomic and morphological data obtained in this study are further supported by a greater in-depth analysis of the genome with a focus on multicellularity, nitrogen fixation and the presence of natural product biosynthetic gene clusters (BGCs). Overall, we here present the reassignment of *Chroococcidiopsis* sp. PCC 6712 to the new *Hyella* species *Hyella disjuncta* PCC 6712.

## 2. Materials and Methods

### 2.1. Origin of Strain and Culture Conditions

The investigated strain was isolated in 1967 by R. Kunisawa from a freshwater reservoir, Marin County, California and originally reported as *Chlorogloea* sp. [5,7,20]. Later on, the strain was re-classified as *Chroococcidiopsis*, but at the same time, the authors noticed significant differences between strain PCC 6712 and other *Chroococcidiopsis* isolates [6].

In our laboratory, the strain was kept in liquid BG11 medium [20] as well as on agar plates in a culture cabinet (CLF Plantclimatics, Percival, Wertingen, Germany) at photosynthetic photon flux density of 30 µmol m^−2^ s^−1^, 20 °C and a light:dark cycle of 16:8 h. The strain was also cultured under the same conditions in 6 well plates filled with 5 g sterilized quartz sand and basalt grains, to which we added 2 mL liquid BG11 medium twice a week to stimulate growth on lithic substrates, known as a preference from other *Hyella* species [16]. Additionally, other liquid media were used such as NM (seawater base supplemented with the minerals of medium BG11 at half strength) [21] that were used in previous studies for, e.g., *H. patelloides* LEGE 07179 [16], in order to enhance comparability of, e.g., morphology. Additionally, nitrogen-free BG11 medium was tested to check for the nitrogen fixing ability of the strain on which growth was monitored over the course of three months.

Motility of, e.g., baeocytes acts as a discrimination feature between unicellular pleurocapsalean and chroococcidiopsidalean taxa [7]. Thus, a motility test was applied by transferring small proportions of the culture on one side of agar plates with BG11 medium. This side of the plate was fully covered with black adhesive tape and placed in a culture cabinet as described above in a way that the uncovered side was directed orthogonally to the light source. Once a week over the course of three months it was checked regularly by means of light microscopy if the cells were motile at any stage of their developmental cycles and moved towards the light.

### 2.2. Morphological Characterization

The morphology of strain PCC 6712 was checked weekly over the course of several months growing in liquid BG11 and NM medium, on BG11 agar plates, on quartz sand and basalt grains by light microscopy using a Panthera KU Trinocular (Motic, Barcelona, Spain) equipped with 10×, 20×, 40× and 100× magnification, oil immersion and an Auramine O fluorescence module (455 nm) coupled with a MicroLive Multi Format camera (Lifesolution, Bremen, Germany) and the software MicroLive (v4.0). In addition, differential interference contrast (DIC) images were taken with an Axisokop (Carl Zeiss, Jena, Germany). Fifty images were taken from the strain and the length and widths of the cells were measured for 50 cells with MicroLive (v4.0). To visualize the sheath material of the cells ACN staining was used (20:1:1 mix; 0.1 g Astrablue in 79.5 mL H_2_O and 2.5 mL acetic acid; 0.1 g Chysoidine in 100 mL H_2_O; 0.1 g Neufuchsin in 100 mL H_2_O; Carl Roth, Karlsruhe, Germany) that allows a differentiation of structures after colors due to the binding characteristics of the substances. Acid mucopolysaccharides are stained blue by Astrablue, cellulose or lignin are stained red by Neufuchsin and hydrophobic substances, such as Cutin, are stained yellow.

### 2.3. Molecular Characterization

The 16S-23S ITS gene region of the strain was sequenced by us to ensure its authenticity after the following workflow. Roughly 20 mg of three weeks old culture material was scraped off agar plates and collected in 1.5 mL tubes with 300 µL SoluLyse, a lysis buffer for protein extraction of bacteria (Amsbio, Abingdon, England). Samples were crushed manually with potter sticks and incubated over night at room temperature on a shaking plate at 110 rpm (Unimax 1010; Heidolph Instruments, Kehlheim, Germany). Afterward, 300 µL of Buffer B (1.4 M NaCl, 20 mM EDTA-Na_2_, 100 mM Tris–HCl, pH 8.6) were added and vortexed for a few seconds. DNA was purified by adding 500 µL chloroform:isoamyl alcohol (24:1), shaking for 5 min and centrifuging for 5 min at 11,000× *g*. The resulting upper phase was transferred to a new 1.5 mL tube and 500 µL of phenol:chlorophorm (1:1) was added. After shaking and centrifuging, the chloroform/isoamyl step was repeated and the supernatant again collected to which 1/10 (*v*/*v*) sodium acetate and 2/3 (*v*/*v*) of isopropanol were added in order to precipitate the extracted genomic DNA in the freezer overnight. Finally, a DNA pellet was obtained by spinning the DNA down at 14,000× *g* for 20 min, which was washed with 500 mL pre-chilled ethanol (70%) and resuspended in 100 µL ddH2O.

The 16S-23S ITS gene region was amplified by PCR in a 50 µL reaction using the primers Wil1 and Wil18 [22] and ready-to-go PCR mini beads (GE Healthcare, Chicago, IL, USA) in a miniAmp thermocycler (Thermo Fisher, Waltham, MA, USA) under the conditions described in [22]. The quality of the PCR products was checked by means of agarose gel electrophoresis using 1% (*w*/*v*) agarose and subsequently purified with the NucleSpin Gel and PCR Clean-up Kit (Macherey-Nagel, Düren, Germany) following the DNA and PCR clean-up protocol. Purified PCR products were sent for Sanger sequencing to Genewiz (Leipzig, Germany) using primers Wil1, Wil4, Wil5, Wil10, Wil11, Wil16 and Wil18.

The generated sequences were assembled with Geneious Prime (v2021.0.1) software package (Biomatters Limited, Auckland, New Zealand) and compared to the genome of the strain that was already generated and deposited by others at NCBI.

The assembled 16S rRNA gene sequences obtained from the isolate and related sequences of cyanobacterial strains cited from GenBank were used for phylogenetic analyses including *Gloeobacter*, representing one of the evolutionary oldest cyanobacterial taxa as an outgroup for the 16S rRNA alignment applying the Muscle algorithm in Mega X v10.2.4 [23].

Finally, 100 nucleotide sequences were used for the phylogenetic comparison, including 1466 bp of the 16S rRNA gene. Ambiguous regions within the alignments were adjusted or removed manually, allowing smaller final blocks and gap positions within the final blocks. The evolutionary model that was best suited to the used database was selected on the basis of the lowest AIC value (Akaike Information Criterion) and calculated in Mega X. The phylogenetic tree was finally constructed with Mega X using the evolutionary Kimura 2 Parameter Model+G+I of nucleotide substitutions. The maximum likelihood method (ML) with 1000 bootstrap replications was calculated with Mega X and Bayesian phylogenetic analyses with two runs of eight Markov chains were executed for one million generations with default parameters with Mr. Bayes 3.2.1 [24]. The analysis reached stationarity (average standard deviation of split frequencies between runs < 0.01) well before the end of the run. Finally, the phylogenetic tree was recalculated with NGPhylogeny.fr [25] and displayed and edited with iTOL v6.3 [26].

Models of the secondary structure of 16S–23S ITS region of all isolates were built in comparison to phylogenetic or morphologically related genera. Helices were folded with the online software RNAstructure Web Server [27].

### 2.4. Holotype Preparation

The new species was described following the rules and requirements of the International Code of Nomenclature for algae, fungi and plants [28]. For the preservation of the type strain a young, 3-week-old culture was transferred into a 5 mL glass bottle with a 4% (*v*/*v*) formaldehyde-water mixture. Preserved material is deposited in the Herbarium Hamburgense, Hamburg, Germany (HBG-025124) while living culture material is available via several public culture collections (PCC 6712, ATCC 27176, CCAP 1411/2).

### 2.5. Bioinformatic Analysis

The genomes of the following organisms were downloaded from NCBI and visualized using the Geneious software package (Version 8.1.9, Geneious, Auckland, New Zealand). All genome comparisons were performed against a database of selected genomes representing the Pleurocapsales (NCBI:txid52604) and *Chroococcidiopsis thermalis* PCC 7203 (CP003597), while other genomes were included as a reference for each specific analysis as described below. 

The selected database of genomes include: *Hyella disjuncta* PCC 6712 (formerly *Chroococcidiopsis* sp. PCC 6712, ANCI00000000); *H. patelloides* LEGE 07179, NZ_LR213766; *C. thermalis* PCC 7203, CP003597; *Myxosarcina* sp. GI1, NZ_JRFE00000000; *Pleurocapsa* sp. PCC 7327 (representative of *Pleurocapsa* sp.), NC_019689; *Pleurocapsales cyanobacterium* LEGE 10410 (representative of *Pleurocapsales cyanobacterium*), JADEXJ000000000; *Stanieria cyanosphaera* PCC 7437, CP003653; *Stanieria* sp. NIES-3757, AP017375 and *Xenococcus* sp. PCC 7305, ALVZ00000000. While *Hyellaceae cyanobacterium* CSU_1_1 and *Hydrococcus rivularis* NIES-593 are classified as Pleurocapsales, they were not included in the analysis due to incomplete data describing their correct taxonomic position. Creation of the genome sequence database, gene cluster alignments and gene cluster figures were generated using the cblaster (Version 1.3.8, Massachusetts Institute of Technology, Cambridge, MA, USA) and clinker (Version 0.0.21, Massachusetts Institute of Technology, Cambridge, MA, USA) bioinformatic tools [29,30]. Default cblaster and clinker settings were used unless otherwise specified.

### 2.6. Genes Involved in Multicellularity

**Figure 1:**
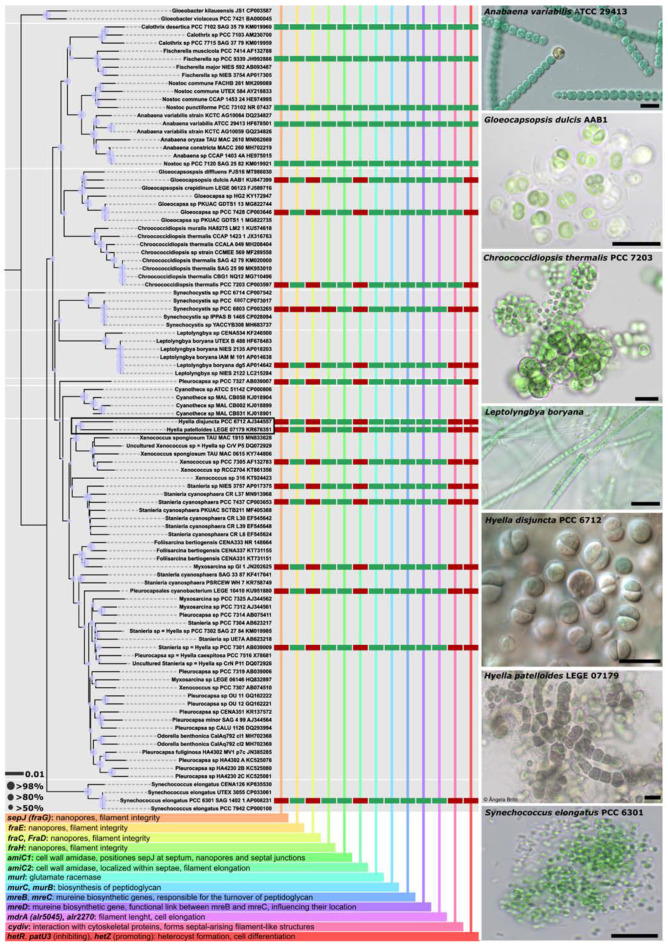
Maximum Likelihood (ML) phylogenetic tree based on the 16S rRNA, overview of genes involved in multicellularity and heterocyte formation and micrographs. The ML phylogenetic tree indicates the position of *Hyella disjuncta* sp. nov. PCC 6712 involving 100 16S rRNA sequences (1466 bp) rooted to *Gloeobacter*. For each sequence, the strain number as well as the NCBI GenBank accession number is displayed. Taxonomic names marked with = were suggested by [3]. Since the resulting Bayesian and ML phylogenetic trees mostly showed the same topology, a single tree with both Bayesian and ML bootstrap values is shown. Statistic support at the nodes (Bayesian inference/Maximum Likelihood) represents posterior probabilities and bootstrap values indicated as circles of different sizes referring to percent intervals. The scale bar specifies 0.01 expected changes per site. The colored chart displays the presence (green)/absence (red) of genes involved in multicellularity and heterocyte formation. Explanations about the gene function are given at the bottom. The right column shows micrographs of isolates (or representatives) whose genomes were investigated in this study. Scale bars are 10 µm.

A list of genes proposed to be involved in multicellularity were first obtained from [31,32], followed by a literature search to include additional genes. For the comparison, we only chose 18 genes with a known function or role involved in multicellularity and the locus_tag of each gene from *Nostoc* sp. PCC 7120 is used as reference. The final list of searched multicellular genes included the *fra* cluster *sepJ* (*fraG*; *alr2338*), *fraE* (*alr2394*), *fraC* (*alr2392*), *fraD* (*alr2393*) and *fraH* (*alr1603*) [33,34,35,36,37,38]; the *amiC* operon genes *amiC1* (*alr0092*), *amiC2* (*alr0093*) and *murI* (*alr0094*) [39,40,41]; the *mur* operon *murB* (*alr5066*) and *murC* (*alr5065*) [42]; *mreB* (*all0087*), *mreC* (*all0086*), *mreD* (*all0085*) [43], *mdrA* (*alr5045*) and *alr2270* [44]; *cydiv* (*all2320*) [45]; and *hetR* (*alr2339*), *patU3* (*alr0101*) and *hetZ* (*alr0099*) [46]. Multicellularity genes listed in Figure 1 were screened using the BLASTp algorithm and each respective gene from *Nostoc* sp. PCC 7120 was used as a query sequence. To be included as a homologue, each gene hit needed to have at least 80% sequence coverage to the query sequence. For alignment of the *ami* gene cluster, BLASTp hits of *amiC1* were used to identify the *ami* operon in each respective genome. The operon was extracted as a genbank sequence and aligned using the clinker tool and with an identity setting (-*i*) of 0.3. Additional genomes involved in multicellularity analysis include: *Fischerella* sp. PCC 9339, ALVS00000000; *Gloeocapsopsis dulcis* AAB1 (formerly *Gloeocapsopsis* sp. AAB1 = 1H9; [47]), NAPY00000000; *Gloeocapsa* sp. PCC 7428, NC_019745; *Nostoc punctiforme* PCC 73102, CP001037; *Nostoc* sp. PCC 7120, NC_003272; *Synechococcus elongatus* PCC 6301, NC_006576; and *Synechocystis* sp. PCC 6308, CM001775. 

### 2.7. Genes Involved in Nitrogen Fixation

Reference *nif* gene clusters were obtained from Thiel et al. [48]. Additional genomes for analysis of nitrogen fixation include: *Crocosphaera subtropica* ATCC 51142, NZ_AGJC02000001; *Leptolyngbya boryana* dg5, NZ_AP014642; *Trichormus variabilis* ATCC 29413 (formerly *Anabaena variabilis* ATCC 29413), NC_007413 and *Nostoc* sp. PCC 7120, NC_003272. The *nifB* gene (based on its essential role in nitrogenase assembly [49]) was searched in all relevant genomes and the cluster extracted as a genbank file. The relevant genbank files were then aligned using clinker and an identity setting (-*i*) of 0.3.

### 2.8. The mys Biosynthetic Gene Cluster

The mycosporine-like amino acids (MAAs) encoded by the *mys* BGC were identified within *Hyella disjuncta* PCC 6712 using the identified locus_tag described in D’Agostino et al. [50]. The entire proposed *mys* operon was used as a query sequence and using cblaster to screen against all available Pleurocasaples genomes in addition to the two *mys* reference genomes of *N. punctiforme* PCC 73102 [51] and *Trichormus variabilis* ATCC 29413 (formerly *Anabaena variabilis* ATCC 29413) [52]. Each operon was extracted as a genbank sequence and aligned using the clinker tool and an identity setting (-*i*) of 0.3.

### 2.9. Genome Mining for Biosynthetic Gene Clusters and Potential Antibiotic Production

The genomes of *H. disjuncta* PCC 6712 and *H. patelloides* LEGE 07179 were submitted to AntiSMASH (Version 6.0, University of Tübingen, Tübingen, Germany) [53] with relaxed settings and all extra features turned on to identify predicted biosynthetic gene clusters (BGCs). The search for possible antibiotic resistance mechanisms and gene duplications was done using the Antibiotic Resistant Target Seeker (ARTS; Version 2.0, https://arts.ziemertlab.com/ accessed on 25 July 2021) [54] with all options turned on.

## 3. Results

The overall results comprising morphology studied under different culture conditions, media and substrates, 16S rRNA based phylogeny and main informative domains of the secondary structures of the 16S–23S ITS sequence led to the conclusion that strain PCC 6712 must be assigned to the genus *Hyella* in the order Pleurocapsales. This is corroborated by in-depth analyses of biosynthetic gene clusters responsible for, e.g., multicellularity and nitrogen fixation obtained from comparisons on genome level; thus, it is now formally described as *H. disjuncta* sp. nov. PCC 6712.

**Figure 2:**
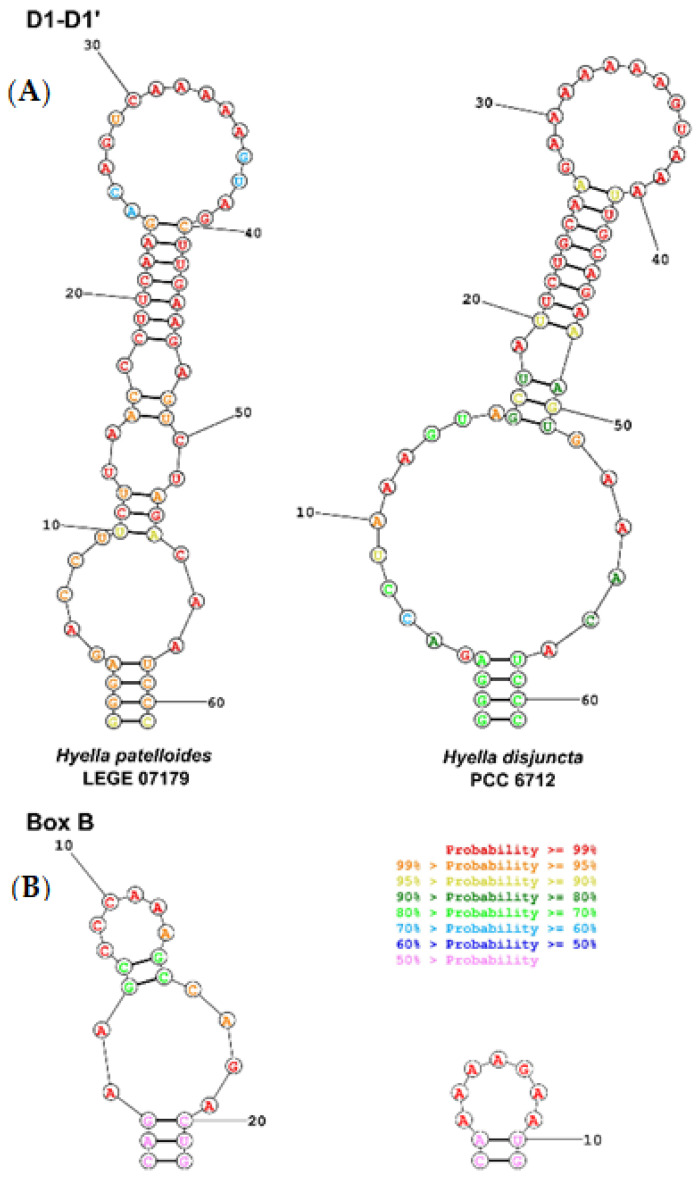
Predicted secondary structures of the 16S-23S rRNA ITS regions of *H. patelloides* LEGE 07179 and *H. disjuncta* PCC 6712. (**A**) D1-D1’ helix, (**B**) Box-B helix.

In detail, *H. disjuncta* PCC 6712 clusters together with *H. patelloides* LEGE 07179 within the order Pleurocapsales based on the 16S rRNA (Figure 1). Related clusters within the Pleurocapsales are formed by *Xenococcus* (Figure 1). The Pleurocapsales represent a monophyletic order significantly different from, e.g., Chroococcidiopsidales or Chroococcales, both orders to which the strain was previously assigned. The D1-D1′ domain within the secondary structures of the 16S–23S ITS indicates a bigger loop structure in the stalk for *H. disjuncta* PCC 6712 compared to *H. patelloides* LEGE 07179 as well as a single loop structure in the Box B domain (Figure 2).

**Figure 3:**
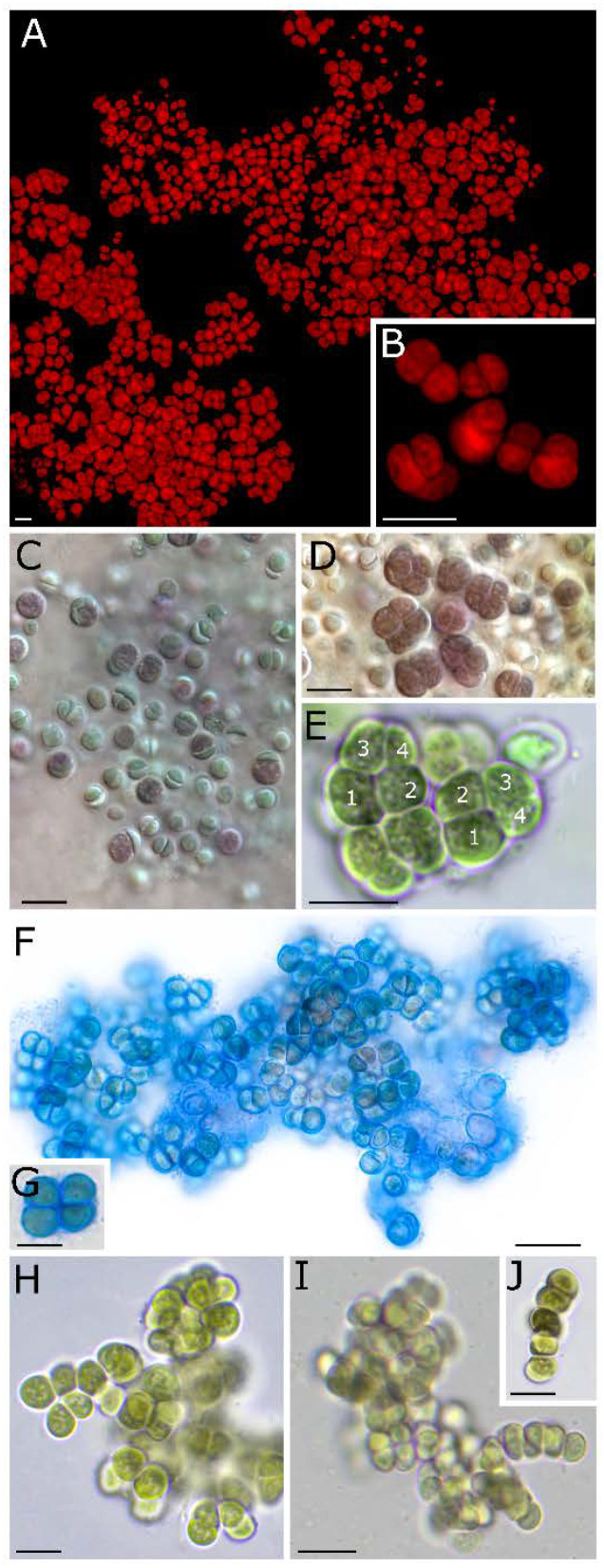
Morphology of *Hyella disjuncta* sp. nov. PCC 6712. (**A**,**B**) Fluorescence microscopy showing the loose, single-cell structure of *H. disjuncta* PCC 6712, as well as a coiled thylakoid structure reaching up to central parts of the cells. (**C**,**D**) Differential interference contrast (DIC) images of ensheathed single cells in (**C**) and baeocytes made of only a few cells in (**D**). (**E**) Baeocyte formation by timed successive binary fission resulting in cells of different sizes corresponding to their age (indicated by numbers). (**F**,**G**) ACN staining showing tight, capsule-like sheath material in blue. (**H**–**J**) Rare formation of short pseudofilaments with cells that are loosely held together by their sheaths.

The morphology of *H. disjuncta* PCC 6712 was found to be stable and uniform during three months of investigations under cultivation in liquid and solidified BG11 and NM medium, on basalt and quartz sand. No growth was detected on nitrogen-free BG11 medium during three months of cultivation; instead, chlorosis could be observed followed by a rapid recovery once transferred to BG11 with nitrogen. *H. disjuncta* PCC 6712 is marked by a loose arrangement of mainly single, unicellular cells of brown to blue-green and purple color (Figure 3). Single cells are enclosed in a colorless, hyaline sheath that is not diffluent and regularly comprises no more than a dozen cells and also baeocytes. Short pseudofilaments were only rarely observed. Cells divide mainly by timed binary fission in more than one successive plane (Figure 3C–E). Reproduction by baeocytes comprising up to eight cells was observed. The strain was found to be non-motile during all life stages. The irregular arrangement of thylakoid membranes is visible already under the light microscope indicated by, e.g., irregular shadings in the cells. The rare formation of short pseudofilaments as well as the single cell formation in *H. disjuncta* sp. nov. PCC 6712 are the main features to discriminate against *H. patelloides*.

### 3.1. Genomic Analysis of Hyella Disjuncta PCC 6712

The genome of *H. disjuncta* PCC 6712 was sequenced and published by Shih et al. 2013 [15] and a comparison with *H. patelloides* LEGE 07179 (as reference genome) was performed by Brito et al. 2020 [14]. Here, we have expanded the genomic analysis of *H. disjuncta* PCC 6712 to include genes involved in multicellularity, nitrogen fixation, the presence of encoded BGCs (AntiSMASH) and potential antibiotic resistance mechanisms (ARTS 2.0). 

#### 3.1.1. Genes Involved in Multicellularity

The genus *Hyella* is characterized by the formation of pseudofilaments, often described as a loose cell–cell contact, where cells are held together mainly by their sheaths, called ‘*articuli disjuncti*’ [55]. While other characterized *Hyella* species form long pseudofilaments, we found these rarely occurring in *H. disjuncta* PCC 6712. Thus, a genomic comparison of *H. disjuncta* PCC 6712 with *H. patelloides* LEGE 07179 and selected species within *Chroococcaceae*, *Synechococcus*, *Gloeocapsa* and *Nostocaceae* were surveyed targeting genes known to be involved in cellular differentiation and multicellularity (Figure 1). In comparison to filament-forming Nostocales, all investigated pleurocapsalean strains including both *H. disjuncta* PCC 6712 and *H. patelloides* LEGE 07179 were highly conserved across all searched genes, missing genes known for their role during multicellularity (*sepJ* (*fraG*), *fraC*, *fraD* and *amiC2*). Interestingly, the *cydiv* gene was present in all Chroococcidiopsidales, such as *Chroococcidiopsis thermalis* PCC 7203, *Gloeocapsopsis dulcis* AAB1, *Gloeocapsa* sp. PCC 7428, all Nostocales and *Pleurocapsa* sp. PCC 7327, but not in any other searched Pleurocapsales. 

**Figure 4:**
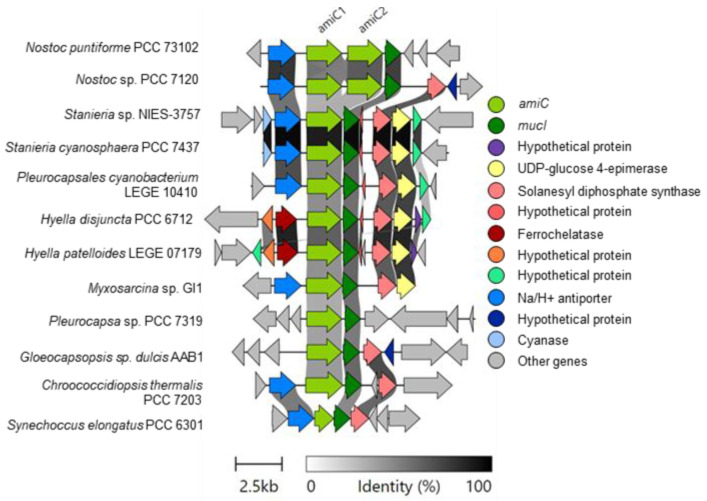
Genomic analysis of the *amiC* operon. Gene similarity cutoff is 0.3 (30%) and cluster alignments are centered using *amiC1*. *Xenococcus* sp. PCC 7305 did encode *amiC1* and *mucI*, but this is not shown because *mucI* is on the edge of a contig.

The *ami* operon has also been found to play a significant role in multicellularity, where Nostocacales appear to encode two copies of the *amiC* (*amiC1* and *amiC2*) gene, while other lineages appear to have a single copy [31]. As expected, both *Hyella* species contained only *amiC1* in addition to the highly conserved glutamine racemace (*mucI*) and a synthase type gene, which has distinct annotations but appeared to have sequence similarity based on clinker alignment. The synthase-type gene was also present in *Nostoc*, *Gleocapsopsis* and *Synechococcus*, but not in *Pleurocapsa* sp. PCC 7319 (Figure 4). Other genes were also conserved within the *Hyella* genus including ferrochelatase, UDP-glucose 4-epimerase (present only in Pleurocapsales) and hypothetical proteins. *Stanieria* sp. and *P. cyanobacterium* LEGE 10410 also showed high similarity to the *Hyella amiC* genetic loci; however, it also encoded an additional Na/H+ antiporter similar to both Nostocales and *Synechococcus elongates* PCC 6301, while this was absent in *Hyella*.

#### 3.1.2. Genes Involved in Nitrogen Metabolism

**Figure 5:**
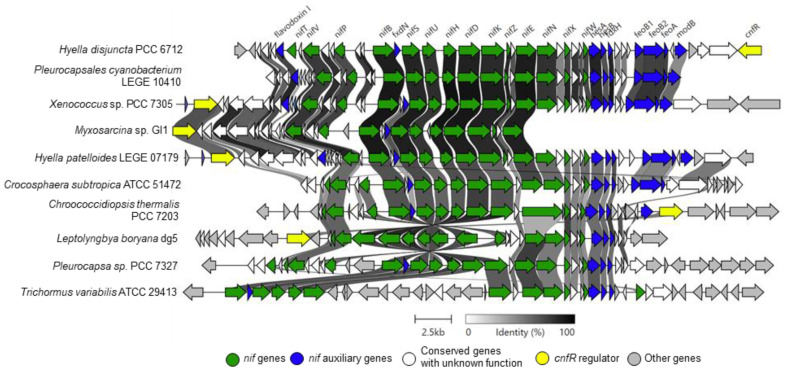
Genomic analysis of the *nif* pathway. Gene similarity cutoff is 0.3 (30%) and cluster alignments are centered using *nifX*.

A genomic screen of the *nif* (nitrogen metabolism) gene cluster was also performed on *Hyella disjuncta* PCC 6712 compared to related organisms. We report here, in agreement with the literature, the inability of the *H. disjuncta* PCC 6712 to grow on nitrogen-deficient medium [5] although we were able to identify the *nif* gene cluster within its genome. The *nif* cluster could not be identified in several other Pleurocapsales genomes, being absent from *Pleurocapsales cyanobacterium* LEGE 10410 and both species of *Stanieria*, while the *nif* cluster was identified in *Myxosarcina* sp. GI1 and showed very similar gene synteny (similar blocks of genes in the same relative positions in the genome) to other Pleurocapsales. However, the cluster was on the edge of a contig and could not be analyzed in full (Figure 5). A comparison of the *nif* gene cluster to other cyanobacteria with well-characterized *nif* pathways showed that genomic synteny of *H. disjuncta* PCC 6712 *nif* appeared most closely to *P. cyanobacterium* LEGE 10410 and not *H. patelloides* LEGE 07179, while *H. patelloides* LEGE 07179 appeared to most closely resemble the *Xenococcus* sp. PCC 7305 *nif* pathway. Furthermore, there was observed diversity between these *nif* clusters, *Pleurocapsa* sp. PCC 7327 and *Chroococcidiopsis thermalis* PCC 7203, particularly in regards to the *feo* genes and the regulator *cnfR* [56] (Figure 5).

#### 3.1.3. Genome Mining Analysis

The genome of *H. disjuncta* PCC 6712 was submitted to AntiSMASH version 6.0 and ARTS 2.0 for detection of BGCs and the possibility of gene duplication as resistance mechanisms. AntiSMASH analysis revealed a total of 13 BGCs (Supplementary Table S1). A total of 10 pathways did not show any similarity to known pathways, two showed very low similarity (<30%) and one showed similarity to the known 1-heptadecene pathway. The 1-heptadecene pathway is responsible for hydrocarbon production by a terminal olefin and was previously identified in other baeocyte-forming strains [57,58]. The 12 cryptic BCGs consisted of three non-ribosomal peptide synthetase/polyketide synthase (NRPS/PKS) hybrid, two type-I PKS, four Lanthipeptide RiPPs, two terpene BGCs and one mixed type-III PKS and terpene BGC. These BGCs are likely to produce a broad range of potentially unknown natural products. 

Analysis of AntiSMASH identified BGCs within the *H. disjuncta* PCC 6712 genome revealed several characteristics. For example, the two BGCs (Region 3.3 and 3.11) encoded halogenases with Region 3.3 encoding four halogenases genes within a single pathway. This pathway shared similarities (although differences in the number of halogenases) to several non-Pleurocapsales cyanobacteria, including representatives of the genera *Chondrocystis*, *Gloeothece*, *Fischerella* and *Microcystis*. Another interesting phenomenon included the high number of glycosyltransferases observed. A total of 5 out of 13 BGCs (Region 3.3, 3.6, 3.7, 3.8 and 3.12) were clustered with glycosyltransferase-like genes, including the PKS-I Region 3.6, which encoded a total of 13 glycosyltransferase-type genes, indicating the likelihood of multiple highly glycosylated natural products. 

Comparison of the *H. disjuncta* PCC 6712 within the AntiSMASH framework was used to identify BGCs that may be present in other cyanobacterial genomes or are likely to be species/strain specific. Interestingly, none of the cryptic BGCs were present in *H. patelloides* LEGE 07179 as a result of the AntiSMASH search, although Brito et al. 2020 [14] found similarities in gene cluster families between the two organisms when using BiG-SCAPE analysis with a 0.7 cut-off which included a single terpene BGC (Region 3.11) and the NRPS-like gene cluster corresponding to the known 1-heptadecene pathway (Region 3.9).

**Figure 6:**
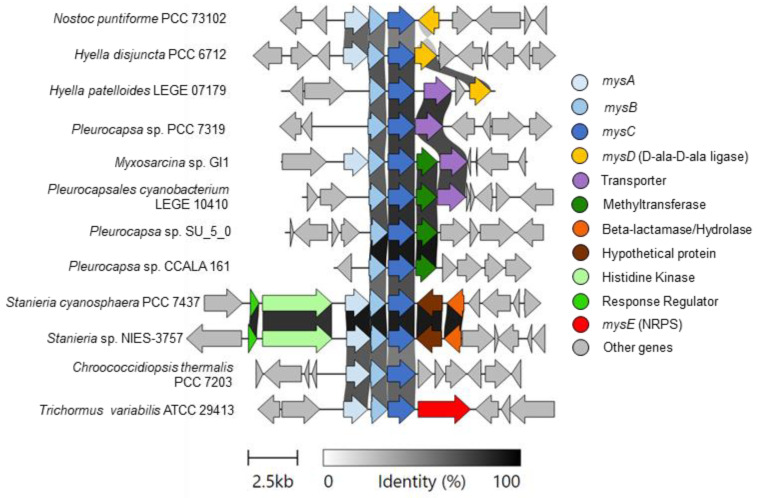
The *mys* gene cluster throughout Pleurocapsales. Gene similarity cutoff is 0.3 (30%) and cluster alignments are centered using *mysC*.

In addition to AntiSMASH-identified BGCs, *H. disjuncta* PCC 6712 has previously been reported to encode the known MAA (UV-absorbing sunscreens) pathway (*mys*) [50], although it was not detected by AntiSMASH. Comparison of the *mys* pathway was performed against all Pleurocapsales, *Chroococcidiopsis thermalis* PCC 7203 and both *Nostoc punctiforme* PCC 73102 and *Trichormus* (*Anabaena*) *variabilis* ATCC 29413 as characterized reference pathways [51,52] (Figure 6). *H. disjuncta* PCC 6712 and *H. patelloides* LEGE 07179 encode distinct *mys* BGCs, yet both form an *N. punctiforme* PCC 73102-type *mys* gene cluster, since they include *mysD* (D-ala-D-ala ligase), in contrast to all other Pleurocaspales and analyzed *mys* pathways, which do not encode *mysD* or *mysE*. The absence of *mysD* and *mysE* from the majority of Pleurocapsales *mys* pathways indicates that these organisms may produce distinct MAAs compared to the most commonly identified shinorine biosynthetic route [50,51], and therefore, they could be interesting to further investigate from biosynthetic and novel molecule perspectives.

*H. patelloides* LEGE 07179 also appeared to contain a *mys* BCG, including two additional genes (a transporter and a GNAT N-acetyltransferase) with unknown biosynthetic function. However, *H. patelloides* LEGE 07179 appears to have a deletion of the *mysA*, which was observed across all other Pleurocaspales with the exception of *Myxosarcina* sp. GI1 and *Stanieria* sp. A BLASTp search using the *H. disjuncta* PCC 6712 *mysA* sequence as a query revealed *mysA*-like homologues in all organisms; however, it is not known if these homologues are involved in MAA biosynthesis.

To further analyze the genome of *H. disjuncta* PCC 6712, we also performed an analysis using ARTS [59], which is used to propose core and resistance genes clustering with BGCs that may indicate possibility of antibiotic production (Supplementary Table S2). ARTS identified 430 core/essential genes and 69 known resistance model hits. A total of 11 BGCs were detected by ARTS. Therefore, there was some discrepancy between AntiSMASH and ARTS BGC identification, where AntiSMASH Region 3.2, 3.5 and 3.13 were not detected by ARTS and one BGC, a bacteriocin, was detected by ARTS and not AntiSMASH. All 11 BGCs were associated with either core resistance (9 out of 11), known resistance (1 out of 11) or putative resistance domains of unknown function (9 out of 11) (Supplementary Table S3).

### 3.2. Taxonomic Treatment

#### *Hyella disjuncta* sp. nov. P. Jung, P.M. D’Agostino, B. Büdel et M. Lakatos

Description: Thallus coherent, dry, brown, rarely violet-brown on agar. It grows as dense, irregular, pseudo-parenchymatous masses in which many cells have a polygonal shape as a result of close packing. Colonies are made of single cells that are more or less spherical, polygonal rounded or oval of highly variable sizes ((3.2)4.6−5.8(7.2) µm in diameter) and a vivid blue-green, purple-violet to brownish color. Cell content is irregularly distributed, including granular inclusions, causing an uneven shading pattern that is also characteristic for *H. patelloides* LEGE 07179. Single cells adhere to colonies that can be irregularly rounded to pseu-do-parenchymatous comprising not more than a dozen cells in a common, hyaline, firm and limited capsule-like sheath. Short pseudofilaments of only a few cells in a raw were exceptionally rarely found, sometimes with two dichotomous apical cells. Cells divide mainly by timed binary fission in more than one successive plane, while reproduction by non-motile baeocytes comprising up to eight cells was observed.

Habitat: unnamed freshwater reservoir, Marin County, California.

Etymology: ‘*disjuncta*’ Latin, describing the mainly unicellular appearance of the species with disjointed cells loosely held together by their sheath, a stadium common to several species within the genus termed ‘*articuli disjuncti*’ by Bornet and Flahaut 1988 [55].

Type location: USA.

Holotype: The preserved holotype specimen of the species is available via Herbarium Hamburgense, Hamburg, Germany (HBG-025124). This was prepared from the living strain.

Reference strain: PCC 6712, synonyms ATCC 27176, CCAP 1411/2, originally reported as *Chlorogloea* sp. [5], originally isolated by R. Kunisawa in 1967.

Discrimination against other species: Despite all other described *Hyella* species, *H. disjuncta* PCC 6712 can be differentiated by being predominantly single-celled, while long pseudofilaments are common for all other species.

Phylogenetic Relations and Secondary Structure of the 16S–23S ITS Sequence: *H. disjuncta* PCC 6712 forms a separate cluster together with *H. patelloides* LEGE 07179 within the Pleurocapsales based on the 16S rRNA phylogeny as well as on genome level. The D1-D1′ domain within the secondary structures of the 16S–23S ITS indicates a bigger loop structure in the stalk for *H. disjuncta* PCC 6712 compared to *H. patelloides* LEGE 07179 as well as a single loop structure in the Box B domain (Figure 2).

Additional Information: Stanier et al. 1971 [20] stated a DNA base composition of 40.8 moles % GC, a maximum tolerable growth temperature of 39 °C and a resistance to penicillin G up to at least 50 units mL^−1^. Bryant 1982 [8] found that the strain produces C-phycoerythrin and exhibits complementary chromatic adaptation of type III (chromatically adapts by photo-controlling the synthesis of both phycoerythrin and phycocyanin). Cumbers et al. 2014 [12] detected salt tolerance up to 500 mM (4%) describing the strain as euryhaline although it is a freshwater isolate and Lorne et al. 2000 [60] found the *kaiC* gene cluster present, a factor involved in controlling the circadian rhythm. The fatty acid profile of the strain was previously analyzed and can be consolidated [10].

## 4. Discussion

### 4.1. Taxonomic Notes on the Genus Hyella

Bornet and Flahault described the genus *Hyella* in 1888 [55], and found several species that often formed polarized pseudofilaments drilling into the carbonate shells of molluscs in marine habitats. Their early findings were based on the species *H. caespitosa;* its complex life cycle and morphology is described in depth by Le Campion-Alsumand and Golubić 1985 [61], ranging from unicellular single celled-stadia, baeocyte formation and H-shaped gelatinous cushion formation between cells up to complex branching patterns of well-expressed pseudofilaments. Comparable morphological characteristics were also described for *H. balani* [61] and *H. patelloides* LEGE 07179 [14]. Besides well-documented morphological comparisons, only a few isolates made their way into public culture collections, allowing a thorough analysis of their taxonomy corroborated by modern phylogenetic tools such as 16S rRNA or whole genome sequencing. In 2017, *H. patelloides* LEGE 07179 was described using the polyphasic approach by Brito et al. [14] without including the 16S rRNA sequence of strain ‘*Pleurocapsa* sp.’ PCC 7516 that was deposited as type strain for the genus as *H. caespitosa* at PCC in 1974 by T. Le Campion-Alsumard, who isolated it from a rock chip at Station B, L’lle Riou, Calanque des Contrebandiers, Marseille, France [7,17]. Our investigations presented here include *H. patelloides* LEGE 07179, the herein described species *H. disjuncta* PCC 6712 and, in addition, the type strain ‘*Pleurocapsa* sp.’ PCC 7516 (*Hyella caespitosa*). A clear relation between *H. disjuncta* PCC 6712 and *H. patelloides* LEGE 07179 in great distance to *H. caespitosa* could be found based on the 16S rRNA what leads to a presumable polyphyly of the genus *Hyella* (Figure 1). We acknowledge this issue because the correct taxonomic evaluation of *H. patelloides* LEGE 07179 and the resulting description considering modern standards is thoroughly conducted, whereas this is missing for the presumed type strain ‘*Pleurocapsa* sp.’ PCC 7516 [62]. Additionally, even on a genome level, phylogeny *H. patelloides* LEGE 07179 and *H. disjuncta* PCC 6712 form a distinct cluster at the same position they can be found in the phylogenetic analysis based solely on the 16S rRNA [14]. In a recent phylogenetic study, the strain *Pleurocapsa* sp. PCC 7516 was integrated as a type strain and some closely clustering 16S rRNA sequences from uncultured and a few cultured strains were considered as additional *Hyella* strains [3]. It should be noted that in their study, the morphology of the strains was not studied and those suggestions were solely based on the cluster formation of the strain’s 16S rRNA sequences [3]. We cannot support these results, but we considered them during our phylogenetic analysis (Figure 1). However, the current polyphyly of the genus will certainly be resolved in the near future when studies combining molecular, morphological and ecological data might re-structure these pleurocapsalean genera and species.

### 4.2. Taxonomic Notes on the Strain Hyella disjuncta PCC 6712, Formerly Chroococcidiopsis sp. PCC 6712

Since its isolation, *H. disjuncta* PCC 6712 has confused taxonomists, which has led to constant taxonomic changes owed to methodological restrictions at that time. It was first classified as the nostocalean *Chlorogloea* sp. [5]. However, it was later noticed that strain PCC 6712 was unable to grow on nitrogen-free medium, an ability that usually all Nostocales share. Based on these doubts and the first genetic analyses, the strain was later placed into the chroococcidiospisidalean genus *Chroococcidiopsis* [6], but morphological differences between *H. disjuncta* PCC 6712 and other *Chroococcidiopsis* strains were noticed that again rang the alarm. Additionally, this misinterpretation was likely also supported by the finding of non-motile baeocytes in strain PCC 6712, which is considered a characteristic feature for non-pleurocapsalean taxa [18,19].

Similarities between *H. disjuncta* PCC 6712 and *H. patelloides* LEGE 07179 can also be found based on morphology, especially during the single-celled stadium of *H. patelloides* LEGE 07179 [16] (Figure 5), which resembles those of the unicellular and single celled *H. disjuncta* PCC 6712. However, both strains can be differentiated by the prominent formation of pseudofilaments of *H. patelloides* LEGE 07179, which remains very weakly and rarely expressed in *H. disjuncta* PCC 6712 (Figure 1) and all other described *Hyella* species. The rather unicellular appearance of *H. disjuncta* PCC 6712 might be confusing because the formation of pseudofilaments was considered to be a strict morphological feature of the genus *Hyella* [55], but unicellular, coccoid stages were already described by Bornet and Flahault in 1888, termed *articuli disjuncti*. They recognized that cells of *H. caespitosa*, the type strain of the genus, were disjointed and can form cellular series rather than trichomes and filaments (=pseudofilaments) and that cells are rather held together only by enclosing sheaths. The coccoid form might be overexpressed in *H. disjuncta* compared to other described *Hyella* species, but this characteristic remained stable since the isolation of the strain and also did not change under various cultivation conditions, such as growth on basalt or sand, as tested in our study over the course of three months. 

Genes responsible for nitrogen fixation were also detected in *H. patelloides* LEGE 07179 [16], but at the same time, they reported no growth on nitrogen-free medium, similar to our findings for *H. disjuncta* PCC 6712, which also encoded the full *nif* cluster (Figure 5). Interestingly, the *nif* cluster was absent from multiple Pleurocapsales. This raises the question if the ability of nitrogen fixation has been lost in all Pleurocapsales or if nitrogen fixation is only possible under specific circumstances, such as during the dark phase of growth or in light at a time when photosynthesis is inhibited [63].

Surprisingly, the formally strain *Chroococcidiopsis* sp. (now *H. disjuncta* PCC 6712) was found to have non-motile baeocytes by Waterbury and Stanier already in 1978 [7] (Figure 3C), which was supported by our findings, questioning if the motility of baeocytes should still be considered as crucial feature reserved for members of the order Pleurocapsales. Unfortunately, it is not known if *H. patelloides* LEGE 07179 forms motile baeocytes or not [16], but the proposed type strain ‘*Pleurocapsa* sp.’ PCC 7516 (*H. caespitosa*) does have motile baeocytes [7], supporting a re-evaluation of the taxonomic status of the proposed type strain ‘*Pleurocapsa* sp.’ PCC 7516 (*H. caespitosa*) or *H. disjuncta* and *H. patelloides* in the future.

### 4.3. Genomic and Chemical Notes on the Strain H. disjuncta PCC 6712

In 2000, the fatty acid profile of many cyanobacterial strains across the phylum, including strain PCC 6712, supported the evidence of taxonomic misassignment and found some overlap to pleurocapsalean strains [10]. This was further elucidated by full 16S rRNA sequencing and phylogenetic analyses in 2001, where a placement within the order Pleurocapsales was supported [4], and has since been linked to the 1-heptadecine BGC (Region 3.9) [14]. The BGC encodes a PKS-I like pathway beginning with a fatty acid ligase, usually known to incorporate a long fatty acid chain [57], followed by a single partially reducing PKS module with domain architecture acyl carrier protein (ACP), ketosynthase, acyltransferase, ketoreductase, a second ACP, sulfotransferase and finally a hydrolase domain. Previous sequence analysis has revealed that this BGC is involved in the production of a terminal olefins by an olefin synthase (OLS) of the CF-8 family [64], with the gene phylogenetically most closely related to *Xenococcus* sp. PCC 7305 and *H. patelloides* LEGE 07179 based on gene cluster family analysis [14,58]. As suggested by Brito et al. and Zhu et al., these phylogenetic and hydrocarbon analysis profiles corresponded with taxonomy, with *H. disjuncta* PCC 6712 producing low levels of polyunsaturated acids while strains of *Chroococcidiopsis* reported high levels polyunsaturated acids [10,57,58]. Fatty acid composition analysis also revealed that C15 hydrocarbon production by *H. disjuncta* PCC 6712 was similar to that of the closely related *H. patelloides* LEGE 07179 and *Xenococcus* but distinct from other members of the Pleurocapsales, such as *Stanieria* sp. [10]. Overall, these data support the assignment of PCC 6712 as a new species of *Hyella* and not *Chroococcidiopsis*.

To further support the morphological and phylogenetic results presented in this study, a genomic approach was used to compare *H. disjuncta* PCC 6712 with related cyanobacteria. A complete genome wide comparison between *H. disjuncta* PCC 6712 and the most closely related *H. patelloides* LEGE 07179 was recently performed by Brito et al. 2020 [14], and found to share 3619 (44.7%) orthologues and 2256 (27.8%) paralogs, while 2229 (27.5%) did not have any similarity. 

While the genomes of the two organisms appear relatively similar, there have been observed differences in the formation of pseudofilaments, where all *Hyella* are capable of pseudofilament formation, but this seems to very rarely occur in *H. disjuncta* PCC 6712. *H. disjuncta* PCC 6712 encoded all multicellular genes present across *Hyella* and closely related Pleurocapsales. Surprisingly, all Pleurocapsales missed the *cydiv* (Cyanobacteria Division) gene, which encodes a conserved hypothetical protein [45]. Recent localization and partial deletion mutant experiments in *Nostoc* sp. PCC 7120 implicated *cydiv* in filamentous cyanobacterial cell division. Furthermore, *cydiv* could not be completely knocked out, indicating it is an essential gene. Interestingly, *cydiv* was present in all investigated Chroococcidiopsidales and Nostocales and Urrejola et al. 2021 [31] already speculated that members of the Chroococcidiopsidales, such as *Gloeocapsopsis dulcis* AAB1, might have lost the ability of multicellularity. However, they assumed that cell–cell interaction is still present during, e.g., dyade or tetrade formation or among baeocytes. This is supported by short pseudofilaments built by linearly arranged cells in *Chroococcidiopsis muralis* [65], a unique finding for Chroococcidiopsidales. However, this information is accurate because the phylogenetic tree prepared by the authors based on the 16S rRNA shows a clear assignment to the genus *Chroococcidiopsis sensu stricto*, although the morphological abnormality of pseudofilaments is intriguing and worth further investigations regarding multicellularity. The lack of genes so far known to be involved in multicellularity in *H. disjuncta* PCC 6712 and *H. patelloides* LEGE 07179 linked to pseudofilament formation in *H. patelloides* LEGE 07179 indicates that unknown genes are certainly involved in multicellularity. Due to their high genomic congruence and the distinct discrimination feature of pseudofilament formation in *H. patelloides* LEGE 07179 and the absence of the latter in *H. disjuncta* PCC 6712 makes both species suitable objects for future investigations of multicellularity.

Genome mining is an important strategy in the discovery of novel and bioactive natural products. Similar to the results of Brito et al. [14] in regards to the presence of strain-specific BGCs encoded within *Hyella* genomes, *H. disjuncta* PCC 6712 was also found to encode unique BGCs that do not seem to be present within the known cyanobacterial genetic data. Interestingly, several pathways were found to encode halogenases and glycosyltransferases. Halogenated and glycosylated natural products have been shown to display high rates of bioactivity and intriguing structural diversity [66,67] and are, therefore, potent targets for drug discovery. Unfortunately, the products of these cryptic pathways remain unknown. Thus, these results support the hypothesis that the *Hyella* genus appears to be a prolific source of cryptic BGCs and potential bioactive natural products. Interestingly, *H. disjuncta* PCC 6712 and *H. patelloides* LEGE 07179 appear to code a very high number of intriguing and unique BGCs, and therefore, this genus is an attractive target for future bioactive natural product discovery.

## 5. Conclusions

The combination of genome mining (e.g., for potential bioactivity, multicellularity, nitrogen fixation, MAA) and polyphasic evaluation comprising morphology, 16S rRNA-based phylogeny and main informative domains of the secondary structures of the 16S–23S ITS domains offers not only information to classify isolated strains within the phylogeny of cyanobacteria, it also gives insides in the functionality of genes between clades and point to specific clades which could be interesting for biotechnology.

Moreover, this study emphasizes that ignoring morphological features and focusing only on genetic- or genome-based phylogenetics to propose new genera and species concepts [68,69] without giving genus and species descriptions of the investigated strains, leads to confusion and frequent revisions of the taxonomic concept. Currently, taxonomic concepts purely based on genetics have not gained acceptance among cyanobacterial taxonomists and, for example, the establishment of type strains solely on genome data has just recently been rejected by the International Committee on Systematics of Prokaryotes (the committee which governs the Prokaryotic Code) [70,71].

The future taxonomic evaluation of additional *Hyella*-like cyanobacterial strains will likely tackle the current discrepancy that is raised between the phylogeny and morphology of the supposed type strain ‘*Pleurocapsa* sp. PCC 7516’ (*Hyella caespitosa*) and *H. patelloides* LEGE 07179/*H. disjuncta* sp. nov. PCC 6712.

## Data Availability

Generated sequences can be found as stated under the species description.

## Supplementary Materials

The following are available online at https://www.mdpi.com/article/10.3390/life11090916/s1, Table S1: AntiSMASH Summary Hits; Table S2: ARTS Core Summary Hits; Table S3: BGC Proximity Analysis; Table S4: Resistance Models; Table S5: Duplication Hits.
